# A novel method for constructing a rat multifactorial tooth wear model: accurate, inexpensive, and easily established

**DOI:** 10.1186/s12903-026-08116-w

**Published:** 2026-03-14

**Authors:** Xingtong Pan, Xinyi Jiang, Xiaoling Wang, Hao Liu, Yunsong Liu

**Affiliations:** 1 Department of Prosthodontics, Peking University School and Hospital of Stomatology, No.22, Zhongguancun South Avenue, Haidian District, Beijing, 100081 PR China; 2 National Clinical Research Center for Oral Diseases, National Engineering Laboratory for Digital and Material Technology of Stomatology, Beijing Key Laboratory of Digital Stomatology, Beijing, PR China; 3 Central Laboratory, Peking University School and Hospital of Stomatology, Beijing, PR China

**Keywords:** Multifactorial tooth wear, Animal model, Sprague–Dawley rat

## Abstract

**Background:**

Tooth wear, a prevalent multifactorial condition, causes oral issues and indirect systemic risks. However, validated animal models for its clinical pathogenic factors are lacking, hindering pathogenesis study and clinical translation. To fill this gap, this study aims to develop and validate an animal model replicating graded molar wear matching clinical features. By regulating mechanical friction duration and chemical erosion intensity, we’ll create a tool for preclinical multifactorial tooth wear research.

**Methods:**

We simulated multifactorial dental tooth wear on the molars of Sprague–Dawley rats using acid etching, thermal stimulation (hot and cold), and hard food. Animal models of different degrees of molar wear were achieved by controlling the duration and superimposition of stimuli. Alveolar bones from the animal models were examined using microcomputed tomography (micro-CT) and histopathological sections to observe the occlusal surface, dental pulp, and periodontal tissues, as well as the distance between the alveolar crest and enamel–cementum junction.

**Results:**

Micro-CT results demonstrated that wear patterns consistent with clinical wear severity classifications were successfully replicated by adjusting the method and duration of stimulation. Compared with the control group (0.20 ± 0.02 mm), the distance between the cementoenamel junction and alveolar crest was significantly higher in the experimental group (0.82 ± 0.02 mm), indicating alveolar bone resorption, which is relevant to multifactorial dental tooth wear. Hematoxylin and eosin staining showed that, compared with the control group, the experimental group exhibited increased inflammation, vacuolar degeneration, dentinal calculus, and formation of reparative dentin in the pulp. The concurrent occurrence of periodontal and pulp lesions during wear aligned with the clinical presentation of wear-associated conditions.

**Conclusions:**

We successfully developed an accurate, inexpensive, easily established model of maxillary molar wear in rats. By controlling the duration and type of stimulation with hard food, hot–cold cycles, and acid etching, we quickly and efficiently constructed models with differing degrees of multifactorial tooth wear suitable for experimental studies and clinical needs.

**Supplementary Information:**

The online version contains supplementary material available at 10.1186/s12903-026-08116-w.

## Background

Dental wear has many causes, including unavoidable mechanical friction during chewing, an acidic diet, medication intake, unconscious tooth clenching while under stress, nighttime tooth grinding, acid reflux, and improper brushing habits [1–3]. Tooth wear is an unavoidable clinical issue that cannot be completely repaired by regeneration with current technology. Mammalian tooth evolution has tended toward hypsodont dentition (i.e., teeth with higher crowns); in humans, this trend is likely attributed to increased lifespan or the necessity to cope with greater exposure to abrasive substances [4]. Thus, the occurrence of tooth wear in humans is increasing, with 30.4% of permanent teeth in children and adolescents now exhibiting tooth wear [5]. With increasing life pressures and changes in dietary habits, the age of patients with tooth wear is gradually decreasing, and its severity in patients of the same age is significantly worse than it was a decade ago [6, 7]. For example, between 2004 and 2005 and 2014–2015, the overall prevalence of tooth wear increased from 31.3% to 45.4%, with more than 50% of adults having at least one tooth surface with moderate wear and approximately 20% having at least one tooth surface at risk of severe wear [4]. A cross-sectional survey of elderly patients in northwestern China revealed the following tooth wear rates in the maxillary jaws of its subjects: 85.51% for molars, 89.77% for premolars, 100.0% for canines, and 87.22% for incisors. In the mandible, the wear rates for the same tooth groups were 86.36%, 88.92%, 100.0%, and 91.19%, respectively [8]. 

Tooth wear leads to defects in enamel and dentin, and affects the morphology of the pulpal cavity, the state of the pulp, and the condition of the periodontium and alveolar bone. These effects include processes such as reparative dentin formation and dental pulp calcification [9]. Patients who are unable to eat or speak normally due to toothache or other effects, such as temporomandibular joint disorders, can suffer from a chain reaction of additional issues such as malnutrition and psychological problems [10]. Studies have shown that severe wear on the occlusal surfaces of teeth caused by betel nut chewing can lead to significant temporomandibular joint disorders, which further exacerbates the physical and psychological burden on patients [11]. With population aging and continued diet refinement, tooth wear will have more serious indirect negative impacts on social stability and economic development [12–15]. Developing appropriate repair plans for different degrees of wear, simulating the therapeutic effects of different repair materials, and studying the pathological mechanisms of wear are all pressing clinical issues.

A previous study constructed caries and wear models by eliminating the oral microbiota of Wistar rats, inoculating them with cariogenic bacteria such as *Streptococcus mutans*, and feeding them a high-sucrose diet [16]. Although this model effectively simulates tooth decay, the accelerated wear caused by such tooth decay does not align with natural wear in its underlying causes or pathological state. Another study used three-dimensional (3D) printing technology to replicate the tooth morphology of Tasmanian devil canines with varying degrees of wear, and then subjected the replicas to penetration tests using a universal testing machine to quantify changes in penetration force/energy attributable to wear [17]. However, the shape and function of canine teeth are very different from those of human molars, which undergo more wear. One study affixed resin attachments to resin models to simulate and study wear associated with braces and determine whether the removal and reinsertion of orthodontic appliances causes wear on tooth surfaces [18]. Despite the clinical significance of such studies, resin differs significantly in hardness from natural teeth, and the frictional forces acting on tooth surfaces during the removal and reinsertion of retainers differ from the chewing forces that cause wear under physiological conditions in their extent, location, and direction. Thus, this method cannot effectively simulate tooth wear under physiological conditions. Studies of the mechanisms, treatment methods, and dental materials related to tooth wear require suitable animal models to support their relevance to human dental wear. However, current animal models for tooth wear have a variety of issues. The lack of integrated models that simulate complex oral environments, acidic diets, and abnormal occlusion represents a significant gap in tooth wear research.

The primary research aim of this study is to develop and validate an accurate, low-cost, and easily reproducible SD rat model of maxillary molar tooth wear that mimics clinically graded wear features (mild, moderate, severe) by regulating the duration and intensity of key pathogenic stimuli (hard food mastication, acid etching, thermal cycling), and to provide a reliable in vivo experimental tool for preclinical research on tooth wear, including studies on its etiology, pathological mechanisms, and evaluation of restorative materials and therapeutic strategies; our initial hypothesis is that regulating the type, duration, and superposition of these key pathogenic stimuli can construct SD rat models of mild, moderate, and severe tooth wear consistent with clinical pathological characteristics, and the constructed models will exhibit periodontal and pulpal lesions (e.g., alveolar bone resorption, inflammatory cell infiltration, reparative dentin formation) corresponding to the severity of tooth wear, which is highly consistent with the clinical manifestations of human multifactorial tooth wear. Therefore, based on the etiological factors of human tooth wear, we developed an accurate, inexpensive, easily established SD rat molar wear model by modulating the duration and intensity of key stimuli, including hard food, acid etchant, and thermal cycling. This model will play a critical role in advancing subsequent research across multiple domains, including the etiology of tooth wear as a multifactorial condition, pathological manifestations of wear induced by distinct causative factors, development and innovation of clinical restorative materials, as well as therapeutic approaches tailored to specific etiologies and severity levels.

## Methods

### Experimental animals

SD rats were obtained from the Animal Testing Center of Peking University Health Science Center. 40 male SD rats (age: 6 weeks, weight: 150–200 g) were housed in a 12-h light–dark cycle at 25 °C in specific pathogen-free animal rooms, with unrestricted access to water and food. Upon arrival, the animals were housed at a density of four rats per cage and underwent a 2-week acclimatization period, with experiments conducted when the rats reached 8 weeks of age. The protocols were approved by the Institutional Animal Care and Use Committee (IACUC) of the Peking University School of Stomatology (no. BDKQ-202501210044).

After adaptive feeding, healthy rats were randomly divided into 5 groups, using a random number table method, with 8 rats per group: control group (normal diet), experimental group 1 (hard food), experimental group 2 (hard food + acid erosion), experimental group 3 (hard food + heat and cold stimulation), experimental group 4 (hard food + acid erosion + heat and cold stimulation). The experimental unit was defined as the individual animal in this study. For each rat, only the right maxillary first molar was selected for observation and measurement, and only one tooth per animal was included in the statistical analysis. All comparisons were performed among independent animals, and no repeated measurement or nested sampling was applied. Animals were excluded from statistical analysis if they presented with severe infection, unexpected death, failure to complete the experimental procedure, or outlier values exceeding the predefined range; all exclusions were documented with clear reasons. The sample size of *n* = 8 per group was determined based on pilot experimental data, previous published studies, and the 3R principles for animal welfare, ensuring adequate statistical power for intergroup comparisons. Humane endpoints were strictly defined: rats were immediately euthanized if they showed severe lethargy, inability to eat or drink, body weight loss exceeding 20%, severe wound infection, or obvious distress. Postoperative monitoring was performed daily to assess the general condition, food intake, activity, and intraoral status of the animals. Additional analgesic and anti-infective treatment was administered as needed to ensure animal welfare.

### Dental tooth wear model

**Fig. 1:**
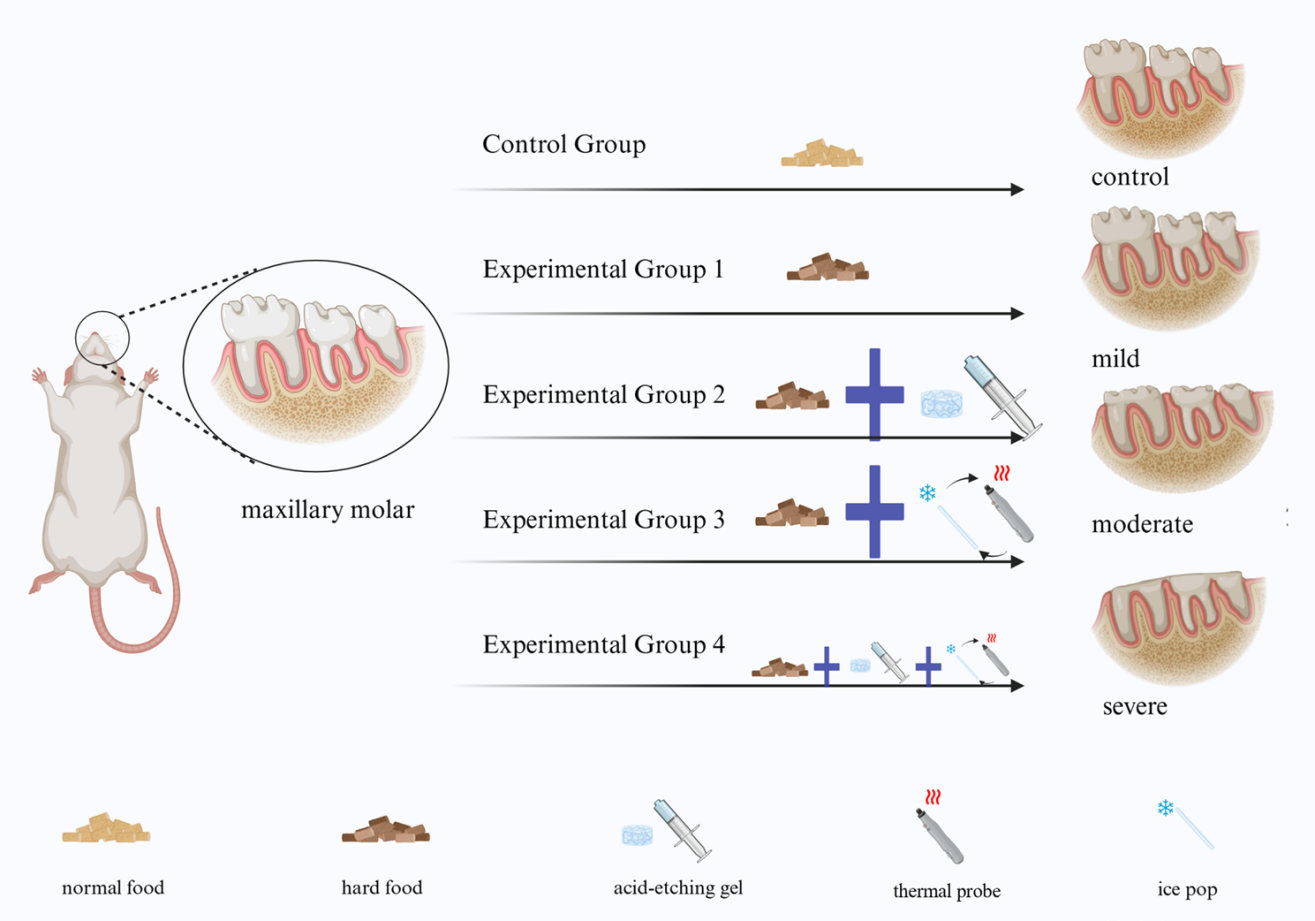
Schematic diagram of the basic steps taken in developing the tooth wear model

Based on a previous study, we selected hard foods, acidic stimuli, and thermal cycling stimuli as physical and chemical factors associated with multifactorial tooth wear [19]. Our experiment modeled different degrees of rat maxillary molar wear by controlling food hardness, the duration of heat (NTE-2 A thermal probe (Jenodi Technology Co., Ltd., Beijing, China)) and cold stimulation (self-made micro ice sticks with a diameter of 0.5 mm were refrigerated at 0 °C for more than 12 h before use), and/or exposure to acid etching gel (GLUMA Etch 35 Gel, Kulzer GmbH, Hanau, Hesse, Germany) (Fig. 1). The real-time temperature of the tooth surface was measured and monitored using the NTE-2 A thermal probe during thermal stimulation.

We used two types of rat feed: Diet HX-3 (Vital River Laboratory Animal Technology, Beijing, China) and Diet MD 17,121 (Mediscience, Jiangsu, China). The feed used in this study was provided in 3 independent batches, and the hardness of intact grains from each batch was measured to ensure consistency. We obtained a 100-g representative sample from each feed bag using the coning and quartering method. From each sample, we selected 20–25 intact grains exhibiting consistent size, length, and structural completeness. Grain hardness was measured using a GWJ-2 grain hardness tester (Jinkelida, Shandong, China) by applying compressive force along the longitudinal axis of each grain. The hardness-testing device was calibrated with standard test blocks before each measurement, following the manufacturer’s instructions. Values from all individual grains were recorded, and the mean hardness was calculated.

Acid etching was conducted as follows: Avertin (tribromoethanol : tert-amyl alcohol = 1:1) (Aibei Biotechnology, Guangdong, China) was diluted to 0.25% in pre-saline and used as a 300-mg/kg intramuscular anesthetic. Following anesthesia, a rat mouthpiece was used to open the oral cavity, and the rat’s incisors were secured. The surfaces of the maxillary molar teeth were swabbed with a cotton ball soaked in 95% alcohol, and then etched with 37% phosphoric acid gel. This gel was applied bilaterally to the surfaces of the maxillary molar teeth and left in place for 10 min, covered by the jaw surfaces. Following treatment, the gel was removed with a cotton ball. The rat was then allowed to awaken naturally.

The heat/cold cycle stimulation steps were as follows: after anesthesia, the surfaces of the maxillary molar teeth were wiped with alcohol-soaked cotton balls, dried, and then treated alternately with a clinical cold test comprising a small ice lollipop (0 °C) and digital constant-temperature heating platform heated to 50 °C. Each temperature was applied for 30 s, and the entire treatment lasted a total of 10 min across five groups. If acid etching gel was used in the experiment, it was removed with a cotton ball. The rat was allowed to awaken naturally.

Due to the greater variety and intensity of stimuli administered to experimental group 4 compared to other groups, the experimental endpoint for this cohort was advanced by 2 weeks to prevent any breach of the replacement, reduction, refinement principle. Half of the animals in experimental group 4 were euthanized 2 weeks post-intervention. Subsequent euthanasia was performed at 4 weeks post-intervention on half of the individuals in experimental groups 2 and 3, and the remaining individuals in group 4. Terminal procedures at the 6-week endpoint included euthanizing all subjects in the control group and experimental group 1, plus the surviving animals in experimental groups 2 and 3 (Fig.S1). Euthanasia was uniformly administered via carbon dioxide inhalation followed by a confirmatory method (cervical dislocation/secondary thoracotomy) in accordance with American Veterinary Medical Association guidelines.

Maxillary molar tooth specimens were collected from euthanized rats and fixed in a 4% paraformaldehyde solution (Servicebio, Hubei, China) for 48 h.

### Microcomputed tomography (micro-CT) assessment

To evaluate intergroup wear differences, the maxillary alveolar bone and molars of the SD rats were scanned and analyzed using an Inveon MM micro-CT system (Siemens, Munich, Germany) following fixation. The scanning parameters were as follows: 80 kV, 500 mA, 360° rotation, 1500-ms exposure time, and a 512 × 512 reconstruction matrix. After scanning, the micro-CT dataset was transferred to a workstation and further analyzed and reconstructed into 3D images using Inveon Research Workplace analysis software (Siemens).

### Pathological staining assessment

Tissues scanned by micro-CT were completely immersed in 0.5-M ethylenediaminetetraacetic acid decalcification solution (Servicebio) and shaken on a shaking platform. The decalcification solution was replaced every other day until there was no resistance to needle penetration. The tissue was cut into 5-µm-thick slices along the sagittal plane and stained with hematoxylin and eosin (H&E) stain. After scanning, the tissue pathology structure was observed using SlideViewer (3DHISTECH, Budapest, Hungary).

### Multifactorial tooth wear grading assessment

We based our assessment of tooth wear in rats on the Smith grading system, which is the most commonly used method for assessing tooth wear in clinical practice [20]. This method uses naked-eye observations to classify remaining tissue based on its quantity and surface morphology. Grade 0 indicates an intact enamel surface with visible developmental grooves and/or only superficial enamel wear. Grade 1 indicates a flat enamel surface with abraded developmental grooves no longer visible, but damage confined to the enamel layer without dentin exposure. Grade 2 indicates localized dentin exposure (≤ 1/3 of the tooth surface) without secondary dentin formation. Grade 3 indicates extensive dentin exposure (> 1/3 of the tooth surface) and/or the presence of secondary dentin. Grade 4 indicates pulp exposure and/or extensive secondary dentin exposure with reduced occlusal vertical distance and/or pulp chamber perforation. On this basis, Grades 0 and 1, which are difficult to differentiate with the naked eye, were grouped as the physiological wear control group. By extension, grade 2 corresponded to mild wear, grade 3 to moderate wear, and grade 4 to severe wear.

All visual grading was performed by two independent examiners blinded to group allocation. Each tooth was scored on a 0–4 scale according to the modified Smith & Knight criteria. To assess the reliability of the scoring, intra-examiner repeatability was tested by one examiner scoring the same set of samples twice with an interval of 3 days. Inter-examiner reproducibility was evaluated by comparing the scoring results of the two examiners. The scoring results are presented in Table S1. The intraclass correlation coefficients (ICC) was calculated to assess intra- and inter-examiner reliability using a two-way mixed-effects model with absolute agreement. The formula for ICC is defined as: $\mathrm{ICC}=\frac{{MS}_{B}-{MS}_{E}}{{MS}_{B}+k(n-1){MS}_{E}+({MS}_{T}-{MS}_{E})\frac{k}{n}}$.

Where MS_B​_ = mean square between subjects, MS_E_​ = mean square error, MS_T​_ = mean square between raters, k = number of raters, n = number of teeth/objects scored.

The intra-examiner and inter-examiner ICC values were calculated according to the formula described above. The intra-examiner ICC was 0.86 and the inter-examiner ICC was 0.89, both of which were higher than 0.85. Therefore, the modified Smith & Knight scoring criteria demonstrated good consistency.

### Statistical analysis

We used GraphPad Prism v9.0 software (GraphPad Software Inc. San Diego, California, United States) for statistical analysis. Data that conformed to a normal distribution were expressed as means ± standard deviation (SD). After comparing means using one-way analysis of variance (ANOVA), we used Tukey’s *post hoc* test to evaluate significant differences at *P* < 0.05. All outcome assessments, including micro-CT image analysis, measurement of crown height and alveolar bone resorption, were performed independently by two trained investigators who were blinded to group allocation to minimize observer bias.

## Results

### Successful construction of a tooth wear model in SD rats using a multi-factor control method

Throughout the experimental period, all subjects reached their respective predetermined humane endpoints. Concurrently, quantifiable molar wear was observed on occlusal surfaces irrespective of treatment assignment.

We quantified the mechanical properties of the two commercially available rodent diets using standardized indentation protocols. The mean hardness values for the two diets were 17.73 MPa (Diet HX-3) and 15.43 MPa (Diet MD 17121), respectively (*P* < 0.001) (Fig. S2). This result validated dietary hardness as a controlled experimental variable distinguishing the intervention and control cohorts.

Subsequently, micro-CT reconstruction of rat dentition demonstrated the successful establishment of molar tooth wear models, with 3D renderings confirming occlusal topographical changes consistent with clinical wear patterns.

**Fig. 2:**
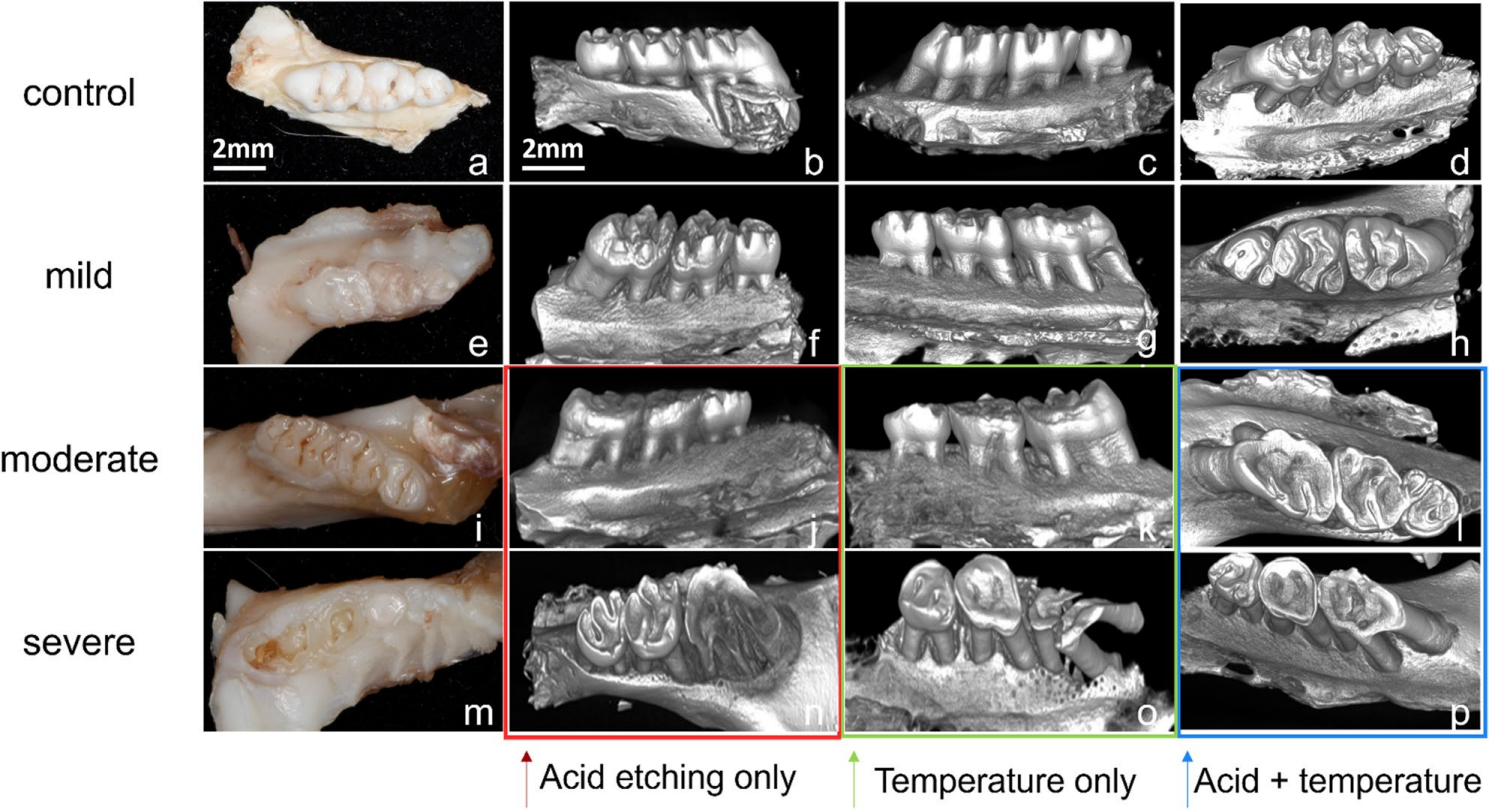
Micro-CT evaluation images of the wear model. **A**-**D** Tooth wear status of tooth tissue surfaces with different degrees of tooth wear after different stimulation treatments. **E**-**H** Wear status of maxillary molar teeth in control rats on a normal diet for 6 weeks. Mild tooth wear of maxillary molars in experimental group 1 rats on a hard food diet for 6 weeks. **I**-**L** Moderate tooth wear of maxillary molars in rats from various experimental groups. **J** Tooth wear status of maxillary molar teeth in rats after 4 weeks of acid erosion plus hard food. **K** Tooth wear status of maxillary molar teeth in rats after 4 weeks of thermal cycling plus hard food. **L** Tooth wear status of maxillary molar teeth in rats after 2 weeks of thermal cycling plus acid erosion plus hard food. **M**-**P** Severe tooth wear of maxillary molars in rats from various experimental groups. **N** Tooth wear status of maxillary molar teeth in rats after 6 weeks of acid erosion plus hard food. **O** Tooth wear status of maxillary molar teeth in rats after 6 weeks of thermal cycling plus hard food. **P** Tooth wear status of maxillary molar teeth in rats after 4 weeks of thermal cycling plus acid erosion plus hard food

Rats in the control group exhibited normal wear on their maxillary molars, characterized by a decreased cusp slope on the occlusal surface. In contrast, rats in the experimental group displayed varying degrees of tooth wear on their maxillary molars (Fig. 2A-D).

Experimental group 1 exhibited enamel loss on the occlusal surface of the maxillary molars, with no significant changes in the morphology of the occlusal surface. This level of wear was classified as mild tooth wear (Fig. 2E-H).

Half of the rats in experimental groups 2 and 3 were treated for 4 weeks, while half in experimental group 4 were stimulated for 2 weeks, during which they experienced partial loss of dentin in their maxillary molars. This level of wear was classified as moderate tooth wear (Fig. 2I-L).

The remaining rats in experimental groups 2 and 3 were treated for 6 weeks, while the remaining half in experimental group 4 were stimulated for 4 weeks, during which a significant amount of dentin was lost and the pulp was exposed. This level of wear was classified as severe tooth wear (Fig. 2M-P).

The maxillary first molar crown height was measured by micro-CT in 8 rats per group. Crown height was defined as the vertical distance from the highest point of the molar crown to the cementoenamel junction (CEJ) plane. The mean crown height was 1.42 ± 0.15 mm in the control group, 1.36 ± 0.06 mm in the mild wear group, 1.13 ± 0.07 mm in the moderate wear group, and 0.65 ± 0.03 mm in the severe wear group. One-way ANOVA followed by Bonferroni post-hoc test revealed a significant difference in crown height among the four groups (*P* < 0.001). Compared with the control group, the crown height was significantly reduced in the moderate and severe wear groups (both *P* < 0.05). Moreover, the severe wear group exhibited a significantly lower crown height than the mild and moderate wear groups (all *P* < 0.05).

These data clearly demonstrate that molar crown height decreased gradually and significantly with the increasing severity of tooth wear, which was highly consistent with the grading results obtained by the modified Smith & Knight visual scoring system.

**Fig. 3:**
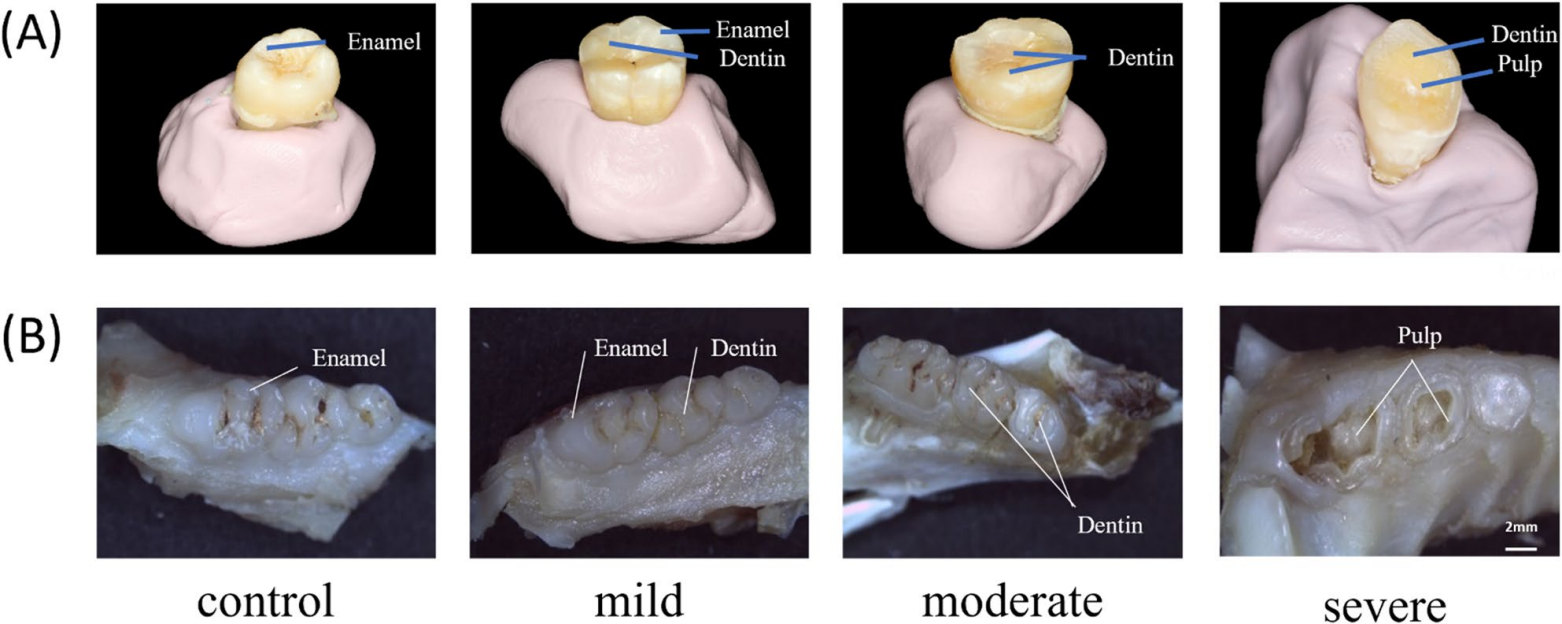
Macroscopic images of varying degrees of wear on (**A**) human and (**B**) rat molars. Bar = 2 mm

These findings align with the outcomes of natural attrition observed in human teeth (Fig. 3).

### Tooth wear induces corresponding damage to periodontal tissues and dental pulp

**Fig. 4:**
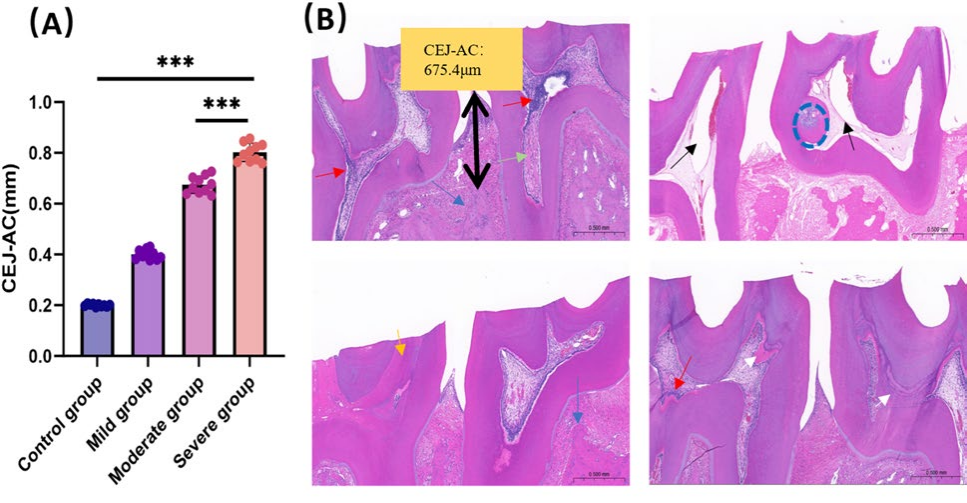
CEJ-AC distance and H&E histology in rat tooth wear models of different severities. **A **Comparison of cementoenamel junction to alveolar crest (CEJ–AC) distances in four wear groups. **B **Hematoxylin and eosin (H&E) staining in the rat tooth wear model. Left panels show representative micrographs from the severe tooth wear cohort; right panels show corresponding specimens from the moderate group. Double-headed arrow，CEJ-AC；blue arrows, alveolar bone resorption; yellow arrows, pulp necrosis; red arrows, pulp inflammation; green arrows, edema; black circles, pulp stones; black arrows, pulpal vacuoles; white arrows, reparative dentin. Bar = 0.5 mm

The distance from the cementoenamel junction (CEJ) to the alveolar crest (AC) is an indicator of periodontal tissue health. In humans, a CEJ–AC distance exceeding 2 mm indicates the presence of alveolar bone resorption [21]. Compared to the control wear group (0.20 ± 0.02 mm), the CEJ–AC distance was significantly higher in the mild (0.40 ± 0.04 mm), moderate (0.68 ± 0.06 mm), and severe (0.82 ± 0.12 mm) wear groups (*P* < 0.001; Fig. 4A).

Histopathological results revealed varying degrees of periodontal and pulpal lesions in rat molars, which were consistent with observations in human teeth. In the control wear group, the tooth and alveolar bone surfaces appeared smooth and intact, with a uniform width in the periodontal ligament space. Almost no inflammatory cell infiltration was observed in the periodontal or pulpal tissues of the control rats. In contrast, the model group exhibited destruction of the tooth surface, reduced alveolar bone height, and pulpal manifestations including inflammatory cell infiltration, vascular dilation, and vacuolar lesions. The severe wear group cohort exhibited pulp exposure, inflammatory cell infiltration, and alveolar bone resorption (Fig. 4B, left panels). The moderate wear group showed characteristic pathological features including pulp stones and reparative dentin formation (Fig. 4B, right panels). These differential pathological signatures correspond to progressive stages of tooth wear degeneration.

## Discussion

Based on clinically established etiological factors for tooth wear, this study introduced common and well-defined pathogenic factors, including acid etching, hard food mastication, and thermal cycling, to establish a wear model in SD rat molars. This model offers high operability, controllable outcomes, readily accessible materials, rapid establishment, and low technical sensitivity. It addresses the lack of a standardized animal model for this prevalent clinical condition, thereby establishing an in vivo experimental foundation for subsequent research.

Variations in clinical restoration approaches and pathological changes in wear are intrinsically linked to their primary causative factors (e.g., acid erosion or hard food chewing). To investigate wear from specific etiologies or evaluate restoration strategies for distinct wear types, our model allows flexible adjustment of single-stimulus parameters (e.g., dosage and duration) to control the dominant pathogenic factor. For example, wear caused by gastric acid reflux can be simulated by increasing the volume of acid etchant applied to the lingual surfaces of target teeth, reducing or eliminating thermal cycling stimuli, and decreasing the proportion of hard food in the diet.

The Smith grading system, which is the most commonly used method for assessing tooth wear in clinical practice, was proposed in 1984 as a clinical visual assessment method [20]. Its core principle involves grading based on the extent of dentin exposure and morphological changes. However, it is limited by its inability to clearly distinguish between grade 0 and grade 1 wear, which theoretically include teeth with no wear. Therefore, we combined grades 0 and 1 to establish a physiological wear control group that is more consistent with clinical judgment criteria.

With the continuous advancement of digital clinical applications and materials science, the future of dentistry promises ever-improving precision, aesthetics, and performance optimization. Currently, digital technology enables the reconstruction of a patient’s original oral morphology through chairside scanning of intraoral soft tissue topography and adjacent and antagonistic tooth morphology [22]. However, tooth wear typically occurs simultaneously in both arches. When pathological wear or tooth loss in the opposing arch disrupts normal occlusion, reconstruction via digital means alone becomes challenging. Instead, reconstruction necessitates combined simulation using cone beam computed tomography, electronic occlusal analysis systems, facebow transfer, or other techniques, which are time-consuming and labor-intensive [23]. The animal model established in this study effectively addresses this clinical scenario of bimaxillary wear by enabling the simulation of single-tooth wear or unilateral jaw wear. Intact antagonistic and adjacent teeth provide excellent anatomical references for worn teeth. In this model, the observed alveolar bone resorption may be attributed to multiple factors generated during the establishment of wear model, including occlusal trauma caused by abnormal occlusal loading, local inflammatory response, physical irritation associated with experimental procedures, and continuous micro-damage to periodontal tissue.

However, our model has limitations. Due to variability in molar structure among individual rats, which may show different adaptations to different stimuli, and the fact that data on the proportions and thicknesses of different parts of the molar are inconsistent, our experiment was unable to precisely quantify and define the degree of wear. Nevertheless, our model is consistent with the common clinical situation in which wear is detected but its severity, and therefore the nature of the subsequent treatment plan, can only be judged based on the remaining tissue. Therefore, despite its shortcomings, our model is relevant for clinical trials.

Furthermore, evaluating novel restorative materials or techniques requires robust animal models. Rats offer significant advantages including low cost, easy accessibility, and suitability for repeated validation. Our model can be used to generate substantial pre-clinical data prior to phase III clinical trials, while filling the need for an in vivo model for this prevalent condition. Consequently, our results exhibit broad applicability and promising prospects for further development.

Nevertheless, the molar anatomy of SD rats is less representative of human teeth than that of beagles or minipigs. As we pursue models with higher fidelity to precise human wear patterns, it will be necessary to incorporate larger animal species or non-human primates into the experimental scope in future.

In future studies, we will focus on further optimizing this model and extending its clinical translational value. First, more precise and automated wear quantification methods will be established by combining micro-CT 3D reconstruction and artificial intelligence image analysis, so as to overcome the current limitation of insufficient quantification accuracy. Second, we will explore the biological mechanisms of hard tissue loss and periodontal remodeling during tooth wear, focusing on related signaling pathways and inflammatory responses, to provide theoretical support for preventive and therapeutic targets. Third, the model will be improved to simulate clinical mixed-type tooth wear and used for long-term evaluation of new restorative materials, bonding systems and minimally invasive treatments. Finally, cross-species comparative studies will be carried out to better match the pathological features of human tooth wear, and promote the transformation of preclinical data into personalized clinical strategies.

## Conclusions

In the present study, we developed various models of tooth wear in rats using cost-effective and readily available materials, including hard food, acid etching gel, small ice lollipops, and digital constant-temperature heating platform. Our experiment yielded a new animal model that allows the experimenter to control the type and duration of pathogenic stimuli as needed to simulate different degrees of abrasion and obtain an appropriate model of rat molar wear. Notwithstanding its limitations, our study establishes a foundational framework with translational implications spanning clinical dentistry, pathological mechanisms of tooth wear, and biomimetic material development through advanced computational workflows.

## Supplementary Information

Supplementary Material 1.

## Data Availability

The data underlying this article will be shared on reasonable request to the corresponding author.

## Abbreviations

Micro-CT: Microcomputed tomography
3D: Three-Dimensional
SD: Sprague–Dawley
H&E: Hematoxylin and Eosin
CEJ: The Cementoenamel Junction
AC: The Alveolar Crest
IACUC: Institutional Animal Care and Use Committee
ICC: Intraclass Correlation Coefficients
