# Supplementary material for: A novel method for constructing a rat multifactorial tooth wear model: accurate, inexpensive, and easily established
Source: BMC Oral Health. 2026 Mar 14;26:714. doi: 10.1186/s12903-026-08116-w (PMC13101339; doi:10.1186/s12903-026-08116-w)
Supplement: Supplementary file 1 — Supplementary Material 1. [file 12903_2026_8116_MOESM1_ESM.docx]

**Supplement figure**


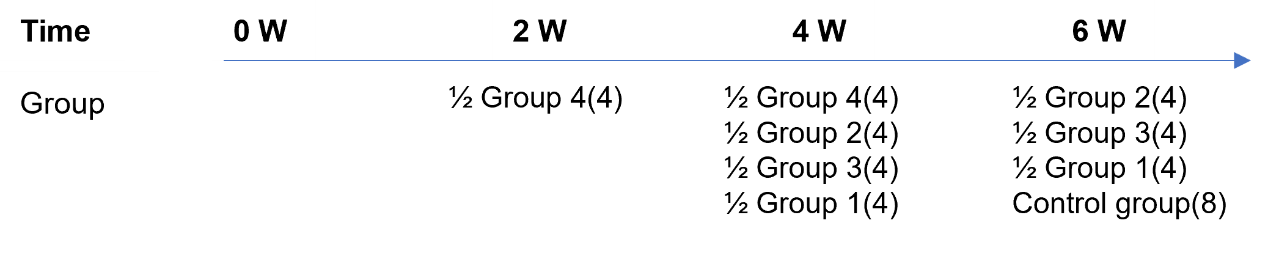


Figure S1 Distribution of the number and timing of euthanasia in animals from different experimental groups. The numbers in parentheses indicate the sample size of each group.


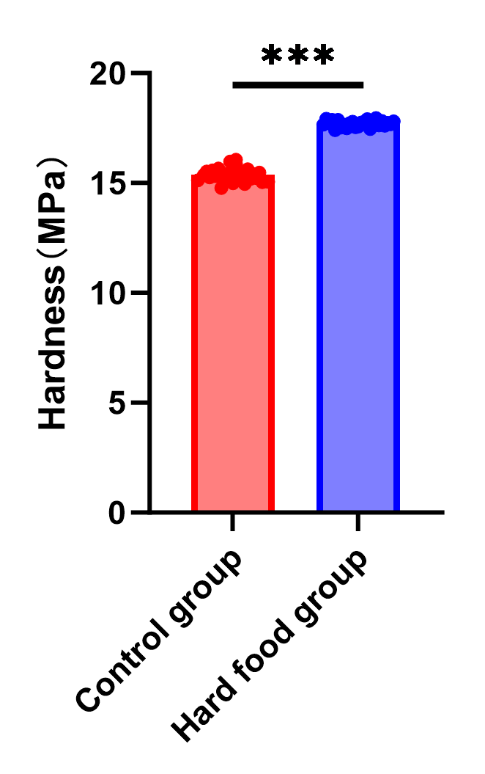


Figure S2 Quantitative analysis of the hardness of rat feed in two groups. (p<0.001)

**Supplement table**

Table S1 Intraclass Correlation Coefficient (ICC) Reliability Analysis Scoring Form

| Sample ID | Examiner A 1st Score | Examiner Re-test Score | Examiner B Score |
| --- | --- | --- | --- |
| 1  2  3  4  5  6  7  8  9  10  11  12  13  14  15  16  17  18  19  20  21  22  23  24  25  26  27  28  29  30  31  32  33  34  35  36  37  38  39  40 | 0  2  1  1  1  1  1  2  3  3  4  4  4  2  3  1  1  0  3  2  3  3  3  1  0  4  4  4  3  3  4  1  4  3  4  4  3  4  3  4 | 1  2  1  1  0  1  2  2  3  3  3  4  4  2  3  0  1  0  3  2  3  2  3  1  1  4  4  4  3  3  4  1  4  3  4  4  3  4  3  4 | 0  2  0  1  1  1  1  2  3  3  4  4  4  2  3  1  1  0  3  2  4  3  3  1  0  4  4  4  3  3  4  1  4  3  4  4  3  4  3  4 |
